# Incidence Rates of Cutaneous Immune-Related Adverse Events in Patients with Lung Cancer: A Systematic Review and Meta-Analysis

**DOI:** 10.3390/curroncol32040195

**Published:** 2025-03-27

**Authors:** Zhihui Yang, Yuanyuan Luo, Ruiqi Lu, Xinqi Liu, Hanyu Liu, Suting Liu, Chen Huang, Jinhui Tian, Lili Zhang

**Affiliations:** 1 School of Nursing, Southern Medical University, No. 1023, South Shatai Road, Baiyun District, Guangzhou 510515, China; zhihui6517@smu.edu.cn (Z.Y.);; 2 Evidence Based Nursing and Midwifery Practice PR China: A JBI Centre of Excellence, No. 1023, South Shatai Road, Baiyun District, Guangzhou 510515, China; 3 Evidence Based Medicine Centre, School of Basic Medical Sciences, Lanzhou University, No. 199, Donggang West Road, Lanzhou 730000, China

**Keywords:** immune checkpoint inhibitors, meta-analysis, cutaneous immune-related adverse events

## Abstract

Objective: Cutaneous immune-related adverse events (cirAEs) represent a prevalent manifestation of adverse reactions linked to immune checkpoint inhibitors (ICIs) therapy, substantially affecting patients’ quality of life. This systematic review and meta-analysis aimed to quantify the pooled incidence of cirAEs in this population and strengthen clinical awareness for early recognition and management. Methods: A comprehensive search of PubMed, Embase, CINAHL, Cochrane Library, CBM, CNKI, and Wanfang databases was conducted from inception to December 2022. Literature that reported the incidence of cirAEs in patients with lung cancer receiving ICIs therapy was included. A meta-analysis was conducted using R software, version 4.4.1 to estimate the pooled incidence of cirAEs, and a random-effects model was used for data synthesis. Begg’s rank correlation and funnel plots were used to assess publication bias. Results: A total of 99 articles involving 23,814 patients with lung cancer receiving ICIs therapy were included, with publication dates ranging from 2012 to 2022. The meta-analysis results reveal that the incidence of cirAEs in patients with lung cancer was 20.26% (95% confidence interval [CI (17.12–23.81)]. Significant differences were observed between all subgroups, including continent, study type, combination therapy, dual ICIs therapy, and diagnostic criteria for cirAEs for Grade 1–2 and Grade 3–4 incidences. Conclusions: The incidence of cirAEs in patients with lung cancer is relatively high, particularly undergoing combined or dual ICIs therapy. To comprehensively characterize cirAEs in patients with lung cancer, large-scale multicenter studies integrating real-world pharmacovigilance data are warranted to establish precise incidence estimates and identify clinically significant risk factors. Implications for clinical practice: This review’s insights aroused clinical staff’s attention and concern about cirAEs, potentially enhancing the quality of life of patients with cancer.

## 1. Introduction

According to Global Cancer Statistics, there has been a rapid increase in the incidence and mortality rates of cancer worldwide [1]. In 2022, approximately 4.82 million new cancer cases were recorded in China [2]. Lung cancer is one of the most lethal cancers in the world [3]. Non-smallcell lung cancer (NSCLC) accounts for approximately 85% of all lung cancer cases and accounts for more than 80% of newly diagnosed lung cancers [4].

Cancer treatment has recently benefited from the introduction of immune checkpoint inhibitors (ICIs), providing a better and longer clinical response [5]. ICIs therapy has already entered clinical practice in the upfront setting either alone (pembrolizumab) or in combination with chemotherapy [6], as well as in locally advanced NSCLC after chemo-radiotherapy [7]. The introduction of ICIs therapy has significantly improved the outcomes of patients with cancer, especially in terms of survival rates. ICIs work differently from standard anticancer therapies. However, their wide application can lead to immunity-related adverse events. Although blocking the negative regulatory signals of T cells may be abnormal, it can also alleviate immunosuppression and enhance normal immune response [8]. The gastrointestinal tract, endocrine glands, skin, and liver are the organs most affected by these adverse events [9]. Adverse events of any grade occur in approximately 30% of patients, and toxic effects of grades 3, 4, or 5 occur in up to 10% of cases of NSCLC [10]. Immune-related adverse events not only affect the patient’s quality of life but can also result in the reduction in or discontinuation of antitumor medications, which may affect the treatment [11,12,13]. Cutaneous immune-related adverse events (cirAEs) appear to be one of the most common forms of immune-related adverse events, including pruritus, rash, skin capillary endotheliosis, oral mucosal lichenoid reaction, Sjögren’s syndrome, bullous pemphigoid, vitiligo, and Stevens–Johnson syndrome [14,15].

Prior research indicates significant variability in cirAEs incidence, with anti-PD-1/PD-L1 therapies showing rates of 30–40% and anti-CTLA-4 treatments exhibiting up to 50% [16]. Another study reported a lower incidence of cirAEs. Among patients receiving CTLA-4 inhibitor therapy, the incidence of cirAEs is 43–45%, compared to approximately 18–23% for those undergoing PD-1 inhibitor treatment [17]. Notably, investigations also found variations in the incidence of cirAEs across cancer types. A systematic review and meta-analysis by Wang et al. [18] encompassing 125 clinical trials with 20,128 patients revealed that the lowest incidence was observed in lung cancer (1.55%; 95% CI, 1.23–1.81%), which was not much different from the highest mean incidence of all adverse events documented in melanoma (1.72%; 95% CI, 1.45–2.27%). Such variation may lead to confusion among health professionals. Conducting a comprehensive analysis of cirAEs incidence can serve as a valuable reference for guiding clinical practice. Other systematic reviews have evaluated immune-related adverse events and the safety of one or more ICI classes [19,20,21]. Moreover, some studies have had limited database searches for a specific period. None of these articles were included in the Chinese articles, which may have led to bias in the incidence of cirAEs. Accurate estimation of cirAEs incidence is critical for developing effective control and prevention programs. Thus, this review aimed to identify the incidence of cirAEs in patients with lung cancer receiving ICIs therapy.

## 2. Methods

This meta-analysis was registered in the PROSPERO (CRD42023446074), and the Preferred Reporting Items for Systematic Reviews and Meta-Analyses (PRISMA) guidelines [22] were implemented for reporting. As this was a meta-analysis review, approval from an ethics committee was not required.

### 2.1. Search Strategy

We searched eight databases, including PubMed, Embase, CINAHL, Cochrane Library, CBM, CNKI, and Wanfang, from inception up to 31 December 2022. A combination of MeSH terms and free-text terms was used. Initial key words included “Neoplasm”, “neoplas*”, “tumor*”, “cancer*”, “malignan*”, “carcinoma*”, “Immunotherapy”, “Immune Checkpoint Inhibitors”, “skin”, “irAEs”, “cirAEs”, “derma*”, and “cut*”. The Medical Subject Headings (MeSH) of each key search term and combinations were explored in every database. Boolean operators, such as ‘AND’ and ‘OR’, were used to search for relevant studies. In addition, a search of gray literature was conducted, including the Virginia Henderson International Nursing Library and Google Scholar. We also found additional articles by searching for relevant published meta-analyses for forward and backward citation tracking of the included studies. In the Supplementary Materials, we have provided a detailed description of the search strategy (see Supplementary File S1), which focuses solely on the study of humans and adults in English and Chinese. A list of references to relevant articles was examined to identify additional articles.

### 2.2. Inclusion and Exclusion Criteria

The criteria were as follows: (1) the participants were patients with lung cancer receiving therapy; (2) reporting data on the incidence rate or risk factors of cirAEs; and (3) the research design included a non-randomized controlled trial (nRCT) (including cross-sectional, case–control, and quasi-experimental studies), cohort studies, and a randomized controlled trial (RCT). The exclusion criteria were as follows: (1) studies that were not in English or Chinese, (2) studies with incomplete data or data that could not be analyzed, and (3) duplicate articles and/or data (selected the most recent article).

### 2.3. Data Extraction and Outcomes

Two reviewers (Yuanyuan Luo and Xinqi Liu) independently screened the literature and extracted data after importing the documents into Excel. The process of literature screening was as follows: excluding duplicate studies, reading the titles and abstracts to exclude clearly irrelevant articles (unrelated to outcome of interest) based on the inclusion criteria, and reading the full text to further determine their suitability. The following data were extracted: study characteristics (author, year, country, language, design, sample size, and diagnostic criteria), characteristics of the participants, drug, cancer stage, incidence rate or risk factors of cirAEs. Any disagreements in the data were resolved by a third partner (Ruiqi Lu).

### 2.4. Quality Assessment

The included studies were independently evaluated for methodological quality by two authors (Yuanyuan Luo and Zhihui Yang) applying the JBI critical appraisal checklist [23] (see Supplementary File S2). If disagreement occurred, the reviewers reached a consensus, with a third reviewer (Ruiqi Lu) resolving disagreements or discussing them within the team if needed. Data synthesis and analysis we used statistical software R, version 4.4.1 (with ggplot2 and forest plot packages). Due to the different measurements used in these enrolled studies, effect size was used to evaluate the incidence rate. Statistical significance was defined as a two-tailed *p* < 0.05. The I^2^ statistics and *p* value were used to assess heterogeneity. If I^2^ ≤ 50% and *p* > 0.1, heterogeneity was considered statistically significant and aggregated using a fixed-effects model. If I^2^ was >50% and *p* < 0.1, a random-effects model was used. Sensitivity analyses were performed to examine the stability of the pooled outcomes, and meta-regression analyses were conducted to explore the relationship between the year of publication and incidence of cirAEs. Subgroup analyses were based on continent, study type, combination therapy group, dual ICIs therapy group, and diagnostic criteria of cirAEs. Funnel plots were used to assess potential publication bias.

## 3. Results

### 3.1. Study Selection

**Figure 1:**
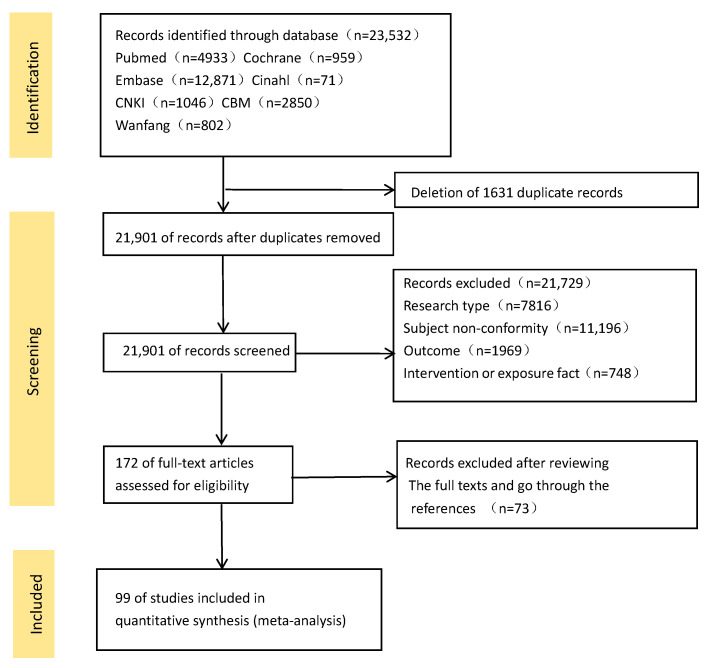
Flowchart of study selection and literature screening process.

The search identified 23,532 relevant studies from nine databases, of which 1631 studies were excluded due to duplication, and 21,729 studies were omitted based on titles and abstracts. Of these, 172 were selected for full-text screening. After reviewing the full texts and reviewing the references, 99 articles [24,25,26,27,28,29,30,31,32,33,34,35,36,37,38,39,40,41,42,43,44,45,46,47,48,49,50,51,52,53,54,55,56,57,58,59,60,61,62,63,64,65,66,67,68,69,70,71,72,73,74,75,76,77,78,79,80,81,82,83,84,85,86,87,88,89,90,91,92,93,94,95,96,97,98,99,100,101,102,103,104,105,106,107,108,109,110,111,112,113,114,115,116,117,118,119,120,121,122] met the criteria. The reasons for exclusion and the process details are shown in Figure 1.

### 3.2. Characteristics of the Included Studies

Across studies, sample sizes ranged from 18 to 1905, totaling 23,814 samples. The incidence rate was 20.26% [95% CI 17.12–23.81)]. Among the 99 studies, 30 were RCTs, 15 were nRCTs, and 54 were cohort studies. The studies were published between 2012 and 2022 and their characteristics are shown in Table 1, Table 2 and Table 3.

### 3.3. Incidence Rate of Cutaneous Immune-Related Adverse Events

**Figure 2:**
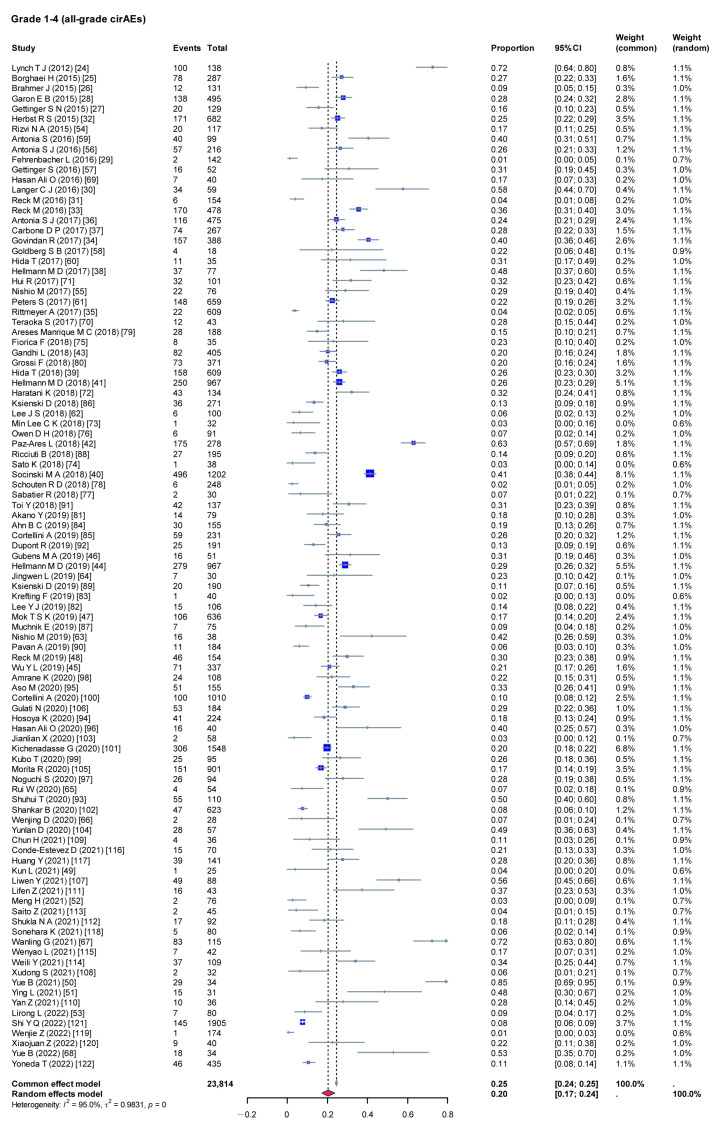
Incidence rate of cirAEs. cirAEs: cutaneous immune-related adverse events; τ: Heterogeneity parameter; I^2^: I-squared statistic; *p*: *p*-value.

A heterogeneity test was performed on the 99 included studies, and the results show high heterogeneity (I^2^ = 95%, *p* < 0.001); therefore, a random-effects model was used to combine the effect values. Meta-analysis results show that the incidence rate of cirAEs was 20.26% (95% confidence interval [CI], 0.1712–0.2381), as shown in Figure 2.

We also analyzed the real-world data from the European Union pharmacovigilance database (EudraVigilance) up to 9 March 2025 for ICIs—including PD-1 inhibitors (pembrolizumab, nivolumab, toripalimab), PD-L1 inhibitors (atezolizumab, durvalumab), and the CTLA-4 inhibitor ipilimumab—commonly used in lung cancer therapy. The aggregated incidence of skin and subcutaneous tissue disorders varied significantly across these agents, with no stratification by specific cancer types. Ipilimumab (Yervoy), a CTLA-4 inhibitor, exhibited the highest incidence rate (15.73%, 2865/18,215 cases). Among PD-1 inhibitors, pembrolizumab (Keytruda) showed a slightly elevated incidence compared to nivolumab (Opdivo) (13.97% [7254/51,928] vs. 13.64% [5654/41,438]), while toripalimab (Loqtorzi) data remained inconclusive due to insufficient reports (0/1 case). PD-L1 inhibitors demonstrated the lowest adverse event rates, with atezolizumab (Tecentriq) at 8.95% (949/10,606) and durvalumab (Imfinzi) at 7.03% (633/8998).

### 3.4. Subgroup Analysis

**Figure 3:**
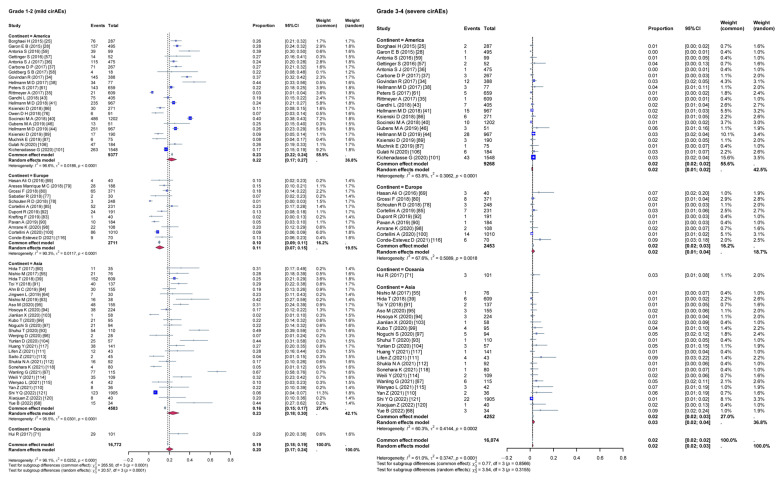
Subgroup analysis (different continent). τ: Heterogeneity parameter; I^2^: I-squared statistic; *p*: *p*-value; χ^2^: Chi-squared statistic; df: Degrees of freedom; Weight Squares: each square represents the effect size of a study, with the size of the square typically proportional to the study’s weight; Diamonds: usually used to represent the summary effect size, with the center of the diamond indicating the estimated summary effect and the width of the diamond representing the confidence interval.

**Figure 4:**
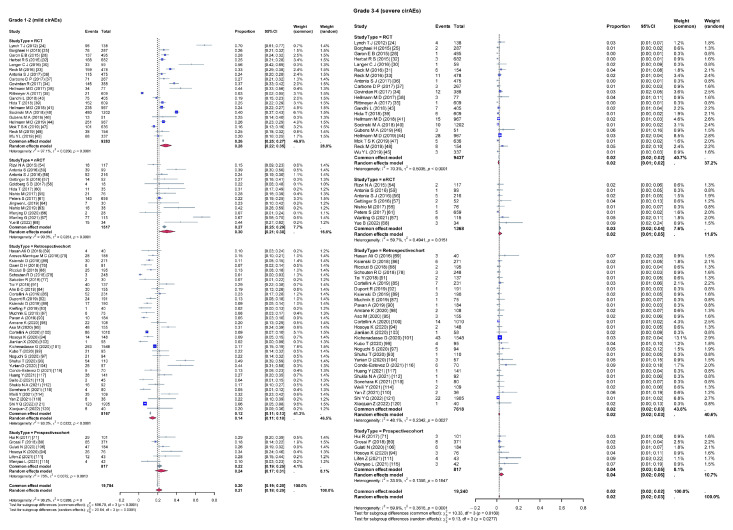
Subgroup analysis (different study type). τ: Heterogeneity parameter; I^2^: I-squared statistic; *p*: *p*-value; χ^2^: Chi-squared statistic; df: Degrees of freedom; Weight Squares: each square represents the effect size of a study, with the size of the square typically proportional to the study’s weight; Diamonds: usually used to represent the summary effect size, with the center of the diamond indicating the estimated summary effect and the width of the diamond representing the confidence interval.

**Figure 5:**
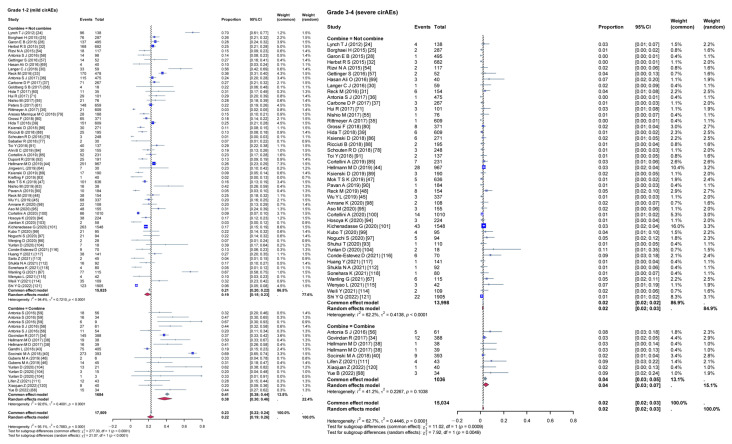
Subgroup analysis (single therapy vs. combined therapy). τ: Heterogeneity parameter; I^2^: I-squared statistic; *p*: *p*-value; χ^2^: Chi-squared statistic; df: Degrees of freedom; Weight Squares: each square represents the effect size of a study, with the size of the square typically proportional to the study’s weight; Diamonds: usually used to represent the summary effect size, with the center of the diamond indicating the estimated summary effect and the width of the diamond representing the confidence interval.

**Figure 6:**
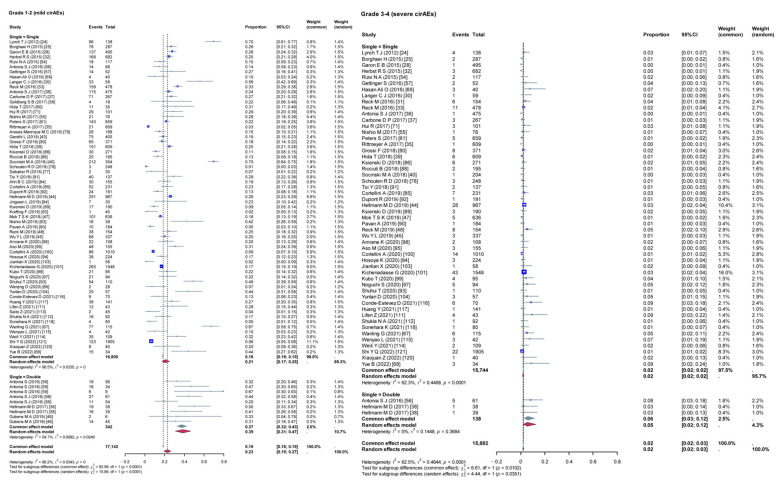
Subgroup analysis (single ICIs therapy vs. dual ICIs therapy). ICIs: immune checkpoint inhibitors; r: Correlation coefficient; τ: Heterogeneity parameter; I^2^: I-squared statistic; *p*: *p*-value; χ^2^: Chi-squared statistic; df: Degrees of freedom. Weight Squares: Each square represents the effect size of a study, with the size of the square typically proportional to the study’s weight; Diamonds: Usually used to represent the summary effect size, with the center of the diamond indicating the estimated summary effect and the width of the diamond representing the confidence interval.

**Figure 7:**
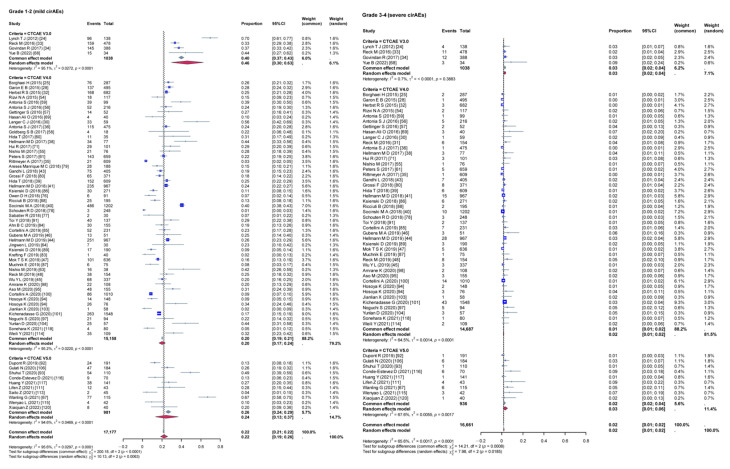
Subgroup analysis (different diagnostic criteria). τ: Heterogeneity parameter; I^2^: I-squared statistic; *p*: *p*-value; χ^2^: Chi-squared statistic; df: Degrees of freedom; Weight Squares: each square represents the effect size of a study, with the size of the square typically proportional to the study’s weight; Diamonds: usually used to represent the summary effect size, with the center of the diamond indicating the estimated summary effect and the width of the diamond representing the confidence interval.

Given the high heterogeneity among the included studies, a subgroup analysis was conducted to evaluate the impact of various variables on the incidence rates of cirAEs. The analysis compared the incidence rates of different grades of cirAEs across subgroups defined by continent (Figure 3), study type (Figure 4), combination therapy (Figure 5), dual ICIs therapy (Figure 6), and diagnostic criteria for cirAEs (Figure 7). As illustrated in Figure 3, Figure 4, Figure 5, Figure 6 and Figure 7, statistically significant differences were observed between all the subgroups for Grade 1–2 and Grade 3–4 cirAE incidences.

### 3.5. Meta Regression

**Figure 8:**
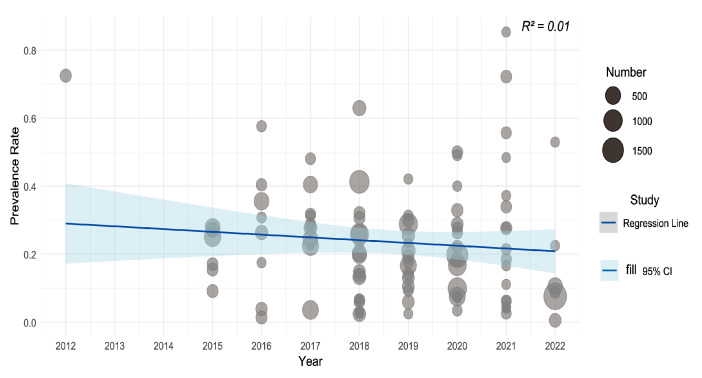
Meta regression Bubble Plot. Figure Note: The size of the circle represents the size of the sample size. The horizontal coordinate is the year. The ordinate is the incidence of cirAEs. cirAEs: cutaneous immune-related adverse events; R^2^: R-squared statistic.

The bubble plot (Figure 8) shows the estimated regression slope for rash incidence and publication time (years). There was no statistically significant relationship between the year of publication and the incidence of cirAEs (R^2^ = 0.01, *p* = 0.2628). The incidence of cirAEs showed a decreasing trend.

### 3.6. Risk Factors for cirAEs in Patients with Lung Cancer Receiving ICIs

Due to the small number of study factors and the large difference in the included risk factors, it was impossible to conduct a combined analysis of the data; therefore, a descriptive analysis of the risk factors of cirAEs was conducted. In terms of demographic data, female patients [93,107] and older patients [80] had a higher incidence of cirAEs. This may be due to damage to the skin barrier structure caused by excessive skincare cleaning, cosmetic use, and excessive use of household chemicals (laundry detergent, dishwashing liquid, etc.) in the female population [93]. Physiological changes and comorbidities in older patients can lead to increased susceptibility to drug-related toxicities [80]. In terms of disease-related data, factors such as cancer stage [93,107], allergy history [93], drug type, and drug combination [93] may influence cirAEs. In patients with advanced tumors (stage II and above), the tumor’s impact and their compromised bodily functions combine to elevate the risk of cirAEs. Patients with a history of allergies are more likely to develop cirAEs after ICIs therapy. CirAEs are more common in patients with a history of chemotherapy. In terms of blood indicators, rheumatoid factors may be risk factors for cirAEs [91,95]. Among patients with pre-existing rheumatoid factors, the incidence of cirAEs was elevated compared to those without it.

### 3.7. Publication Bias

**Figure 9:**
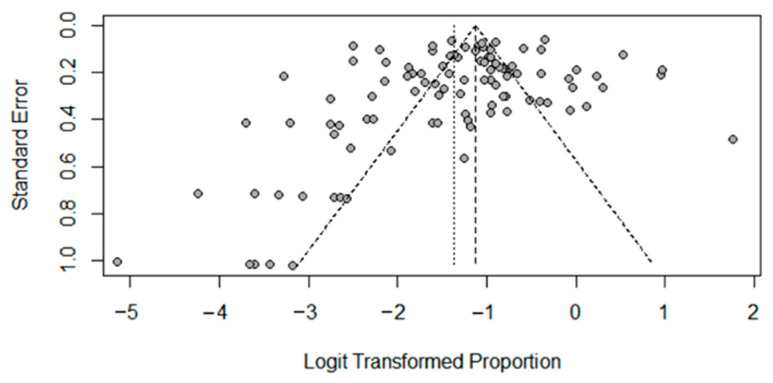
Begg’s funnel plot.

The Egger test was used to check the publication bias of the included literature, and the test results show that the difference was not statistically significant (t = 1.46, *p* = 0.1478), and the scatter distribution in the funnel plot (Figure 9) was basically symmetrical and uniform, so it could be considered that there was no publication bias.

## 4. Discussion

The incidence of cirAEs in patients receiving ICIs therapy is high and should be given sufficient attention. A total of 99 studies published between 2012 and 2022 were included in this study, and the incidence and risk factors of ICIs therapy were described through a systematic review and meta-analysis. The overall incidence of cirAEs was 20.26%. A similar incidence was reported in another study. A systematic review of cirAEs reported an incidence of 20.8% among NSCLC patients, with rash and pruritus occurring rates at of 12.4% and 10.4%, respectively [123]. Meta-analysis enhances drug safety evaluation by aggregating data across studies, compensating for the limited sample sizes of individual clinical trials. However, key challenges include the following: differing incidence rates of adverse events across studies, the possible infrequency of specific target adverse events, and incomplete or inconsistent reporting of adverse events, especially when event counts were below predefined thresholds. Excluding such censored data can bias incidence estimates, necessitating careful handling to ensure accurate statistical inferences [124].

ICIs therapy has achieved remarkable progress in lung cancer treatment, significantly extending patients’ survival time. However, with its widespread use, cirAEs have emerged as a major adverse effect of ICIs therapy. To date, no conclusive evidence has established an association between the clinical manifestations of cirAEs and primary tumor types. Current consensus predominantly attributes these events to drug-related toxicity stemming from the pharmacological properties of therapeutic agents. Some scholars speculate that this may be due to the abnormal targeting of dermal antigens by reactivated T cells and inflammation after cross-reaction with normal skin [125,126]. However, the specific mechanism remains under investigation. With the development of ICIs therapy, an increasing number of scholars have found that the appearance of cirAEs does not seem to be detrimental.

A meta-analysis published in JAMA Dermatology showed that an association was observed between the existence of cirAEs and improved cancer prognosis among patients receiving ICIs therapy. These data suggest that cirAEs may have useful prognostic value in ICIs therapy. More large-scale prospective studies are needed to validate and establish the association between cirAEs and survival outcomes [127]. CirAEs indicate a better prognosis, which may be related to the improvement in body immunity [27,31,35,70]. According to Indini et al. [128], the development of immune-related adverse events during treatment demonstrates a strong correlation with significantly improved progression-free survival and overall survival in patients with metastatic melanoma. Notably, among various adverse events, vitiligo occurrence exhibited a specific association with enhanced overall survival outcomes. Therefore, cirAEs may also reflect the treatment effect. However, this has not been observed in lung cancer. In view of the high incidence of cirAEs in patients with ICIs therapy, the attention of clinical staff should be further improved, relevant risk factors should be identified, and active measures should be taken to prevent the occurrence and development of cirAEs, to avoid progression to severe cirAEs, resulting in forced changes in the treatment program, or even interruption.

The meta-analysis revealed significant heterogeneity among the studies. Further subgroup analysis showed that the heterogeneity may be mainly due to differences in the research methodology and diagnostic criteria. Methodological heterogeneity was due to differences in the sample size, exclusion and inclusion criteria, age distribution, and drugs used in the various original studies. These differences may lead to significant variations in the prevalence of cirAEs. Another heterogeneity was mainly due to differences in the measurement tools, which lead to variations in the grades of cirAEs. Currently, the CTCAE scale is a commonly used assessment tool for cirAEs. However, frequent updates to its version and changes in standards over time have led to discrepancies in the findings. Additionally, the frequency and time of evaluation of cirAEs varied in each study. CirAEs exhibit dose-dependence. Short evaluation periods or infrequent assessments may lead to underestimation of their incidence, thereby causing high outcome heterogeneity. To avoid significant heterogeneity among studies and reduce publication bias, it is recommended to establish uniform standards for future studies. This will facilitate the clinical monitoring of the occurrence of cirAEs and improve the comparability between studies.

### 4.1. Limitations

This study has various limitations that should be acknowledged. First, this study solely queried the EudraVigilance database without integrating real-world data from other pharmacovigilance databases into the comprehensive assessment of cirAEs incidence, which may limit the validity of the findings. Second, although the statistical results from Figure 9 indicate no publication bias, this meta-analysis of adverse event incidence may be subject to potential overestimation bias due to selective reporting, which could influence the pooled estimates of circulating cirAEs in this review. Additionally, cirAEs exhibit a time-dependent pattern—their incidence increases with prolonged treatment duration and cumulative drug exposure. However, inconsistent definitions of “cirAEs initiation time” across studies created uncertainty in evaluating dose–response relationships. Third, significant methodological heterogeneity in diagnostic tools, monitoring frequency, and follow-up durations (e.g., variations in CTCAE versions) persisted despite subgroup analyses. Furthermore, language barriers limited inclusion to Chinese and English literature, potentially omitting critical evidence from other languages. Notably, the research team’s affiliation with the JBI Center for Evidence-Based Practice predisposed us to favor JBI assessment tools, which may affect methodological objectivity.

### 4.2. Implications for Further Research

Future studies on patients receiving ICIs therapy should provide a comprehensive classification of cirAEs and a longer documentation period to better understand their occurrence and patterns. Moreover, future studies must explore the factors that influence cirAEs and their correlation with other immune-related adverse events.

## 5. Conclusions

This study’s results indicate that patients with lung cancer who use immune checkpoint inhibitors are more likely to experience grade 1 and 2 cirAEs compared to those with grades 3 and 4. Combination therapy and dual ICIs therapy can increase the incidence of cirAEs in patients with lung cancer. Regular follow-up and identification of risk factors can help manage and reduce the symptoms of these adverse events. To further explore the incidence and risk factors of cirAEs on patients receiving ICIs therapy, a large-scale, multi-center study is recommended. Health professionals should pay close attention to patients with cirAEs after ICIs therapy and take the necessary measures to manage and reduce the incidence of such events.

## Figures and Tables

**Table 1:** Characteristics of included randomized controlled trials (RCTs).

No.	Author (Year)	Countries	Continent	Language	Design	Sample Size	Diagnostic Criteria	Incidence Rate
1	Lynch TJ (2012) [24]	NA	NA	English	RCT	138	CTCAE V3.0	72.46%
2	Borghaei H (2015) [25]	USA	America	English	RCT	287	CTCAE V4.0	27.18%
3	Brahmer J (2015) [26]	USA	America	English	RCT	131	/	9.16%
4	Gettinger SN (2015) [27]	USA	America	English	RCT	129	CTCAE V3.0	15.50%
5	Garon EB (2015) [28]	USA	America	English	RCT	495	CTCAE V4.0	27.88%
6	Fehrenbacher L (2016) [29]	USA	America	English	RCT	142	CTCAE V4.0	1.41%
7	Langer CJ (2016) [30]	USA, Taiwan	NA	English	RCT	59	CTCAE V4.0	57.63%
8	Reck M (2016) [31]	NA	NA	English	RCT	154	CTCAE V4.0	3.90%
9	Herbst RS (2016) [32]	Argentina, Australia, Belgium, Brazil, Canada, Chile, Czech Republic, Denmark, France, Germany, Greece, Hungary, Italy, Japan, Lithuania, The Netherlands, Portugal, Russia, South Africa, Republic of Korea, Spain, Taiwan, UK, USA	NA	English	RCT	682	CTCAE V4.0	25.07%
10	Reck M (2016) [33]	USA, Australia, Korea	NA	English	RCT	478	CTCAE V3.0	35.56%
11	Govindan R (2017) [34]	USA	America	English	RCT	388	CTCAE V3.0	40.46%
12	Rittmeyer A (2017) [35]	USA	America	English	RCT	609	CTCAE V4.0	3.61%
13	Antonia SJ (2017) [36]	USA	America	English	RCT	475	CTCAE V4.0	24.42%
14	Carbone DP (2017) [37]	USA	America	English	RCT	267	/	27.72%
15	Hellmann MD (2017) [38]	USA	America	English	RCT	77	CTCAE V4.0	48.05%
16	Hida T (2018) [39]	Japan, North America, South America, Europe, and Asia	Asia	English	RCT	609	CTCAE V4.0	25.94%
17	Socinski MA (2018) [40]	USA	America	English	RCT	1202	CTCAE V4.0	41.26%
18	Hellmann MD (2018) [41]	USA	America	English	RCT	967	CTCAE V4.0	25.85%
19	Paz-Ares L (2018) [42]	NA	NA	English	RCT	278	CTCAE V4.0	62.95%
20	Gandhi L (2018) [43]	USA	America	English	RCT	405	CTCAE V4.0	20.25%
21	Hellmann MD (2019) [44]	USA	America	English	RCT	967	CTCAE V4.0	28.85%
22	Wu YL (2019) [45]	China, Russia, Singapore	NA	English	RCT	337	CTCAE V4.0	21.07%
23	Gubens MA (2019) [46]	USA	America	English	RCT	51	CTCAE V4.0	31.37%
24	Mok TSK (2019) [47]	Argentina, Brazil, Bulgaria, Canada, Chile, China, Hong Kong Special Administrative Region, Colombia, Czech Republic, Estonia, Guatemala, Hungary, Japan, Latvia, Lithuania, Malaysia, Mexico, Peru, Philippines, Poland, Portugal, Romania, Russia, South Africa, Republic of Korea, Sweden, Switzerland, Taiwan, Thailand, Turkey, Ukraine, Vietnam	NA	English	RCT	636	CTCAE V4.0	16.67%
25	Reck M (2019) [48]	NA	NA	English	RCT	154	CTCAE V4.0	29.87%
26	Li K (2021) [49]	China	Asia	Chinese	RCT	25	/	4.00%
27	Bai Y (2021) [50]	China	Asia	Chinese	RCT	34	CTCAE V3.0	85.29%
28	Lv Y (2021) [51]	China	Asia	Chinese	RCT	31	CTCAE V4.0	48.39%
29	He M (2021) [52]	China	Asia	Chinese	RCT	76	/	2.63%
30	Li LR (2022) [53]	China	Asia	Chinese	RCT	80	/	8.75%

NA: Not Available; CTCAE V: Common Terminology Criteria for Adverse Events Version; “/”: indicate that specific diagnostic criteria was not adopted in the study.

**Table 2:** Characteristics of included non-randomized controlled trials (nRCTs).

No.	Author (Year)	Countries	Continent	Language	Design	Sample Size	Diagnostic Criteria	Incidence Rate
1	Rizvi NA (2015) [54]	France, Germany, Italy, USA	NA	English	nRCT	117	CTCAE V4.0	17.09%
2	Nishio M (2016) [55]	Japan	Asia	English	nRCT	76	CTCAE V4.0	28.95%
3	Antonia SJ (2016) [56]	Finland, Germany, Italy, Spain, UK, USA	NA	English	nRCT	216	CTCAE V4.0	26.39%
4	Gettinger S (2016) [57]	USA, Canada	America	English	nRCT	52	CTCAE V4.0	30.77%
5	Goldberg SB (2016) [58]	USA	America	English	nRCT	18	CTCAE V4.0	22.22%
6	Antonia S (2016) [59]	USA	America	English	nRCT	99	CTCAE V4.0	40.40%
7	Hida T (2017) [60]	Japan	Asia	English	nRCT	35	CTCAE V4.0	31.43%
8	Peters S (2017) [61]	USA	America	English	nRCT	659	CTCAE V4.0	22.46%
9	Lee J S (2018) [62]	Republic of Korea	Asia	English	nRCT	100	CTCAE V4.0	6.00%
10	Nishio M (2019) [63]	Japan	Asia	English	nRCT	38	CTCAE V4.0	42.11%
11	Li JW (2019) [64]	China	Asia	Chinese	nRCT	30	CTCAE V4.0	23.33%
12	Wang R (2020) [65]	China	Asia	Chinese	nRCT	54	/	7.41%
13	Deng WJ (2020) [66]	China	Asia	Chinese	nRCT	28	CTCAE	7.14%
14	Gan WL (2021) [67]	China	Asia	Chinese	nRCT	115	CTCAE V5.0	72.17%
15	Bai Y (2022) [68]	China	Asia	Chinese	nRCT	34	CTCAE V3.0	52.94%

NA: Not Available; CTCAE V: Common Terminology Criteria for Adverse Events Version; “/”: indicate that specific diagnostic criteria was not adopted in the study.

**Table 3:** Characteristics of included cohort studies.

No.	Author (Year)	Countries	Continent	Language	Design	Sample Size	Diagnostic Criteria	Incidence Rate
1	Hasan AliO (2016) [69]	Switzerland	Europe	English	Retrospective cohort	40	CTCAE V4.0	17.50%
2	Teraoka S (2017) [70]	Japan	Asia	English	Prospective cohort	43	CTCAE V4.0	27.91%
3	Hui R (2017) [71]	Australia	Oceania	English	Prospective cohort	101	CTCAE V4.0	31.68%
4	Haratani K (2018) [72]	Japan	Asia	English	Retrospective cohort	134	CTCAE V4.0	32.09%
5	Min Lee CK (2018) [73]	California, USA	America	English	Retrospective cohort	32	/	3.13%
6	Sato K (2018) [74]	Japan	Asia	English	Prospective cohort	38	CTCAE V4.0	2.63%
7	Fiorica F (2018) [75]	Italy	Europe	English	Retrospective cohort	35	CTCAE V3.0	22.86%
8	Owen DH (2018) [76]	USA	America	English	Retrospective cohort	91	CTCAE V4.0	6.59%
9	Sabatier R (2018) [77]	France	Europe	English	Retrospective cohort	30	CTCAE V4.0	6.67%
10	Schouten RD (2018) [78]	The Netherlands	Europe	English	Retrospective cohort	248	CTCAE V4.0	2.42%
11	Areses Manrique MC (2018) [79]	Spain	Europe	English	Retrospective cohort	188	CTCAE V4.0	14.89%
12	Grossi F (2018) [80]	Italy	Europe	English	Prospective cohort	371	CTCAE V4.0	19.68%
13	Akano Y (2019) [81]	Japan	Asia	English	Retrospective cohort	79	CTCAE V4.0	17.72%
14	Lee YJ (2019) [82]	Korea	Asia	English	Retrospective cohort	106	CTCAE V4.0	14.15%
15	Krefting F (2019) [83]	Germany	Europe	English	Retrospective cohort	40	CTCAE V4.0	2.50%
16	Ahn BC (2019) [84]	Republic of Korea	Asia	English	Retrospective cohort	155	CTCAE V4.0	19.35%
17	Cortellini A (2019) [85]	Italy	Europe	English	Retrospective cohort	231	CTCAE V4.0	25.54%
18	Ksienski D (2019) [86]	Canada	America	English	Retrospective cohort	190	CTCAE V4.0	10.53%
19	Muchnik E (2019) [87]	USA, Canada	America	English	Retrospective cohort	75	CTCAE V4.0	9.33%
20	Ricciuti B (2019) [88]	NA	NA	English	Retrospective cohort	195	CTCAE V4.0	13.85%
21	Ksienski D (2019) [89]	Canada	America	English	Retrospective cohort	271	CTCAE V4.0	13.28%
22	Pavan A (2019) [90]	Italy	Europe	English	Retrospective cohort	184	/	5.98%
23	Toi Y (2019) [91]	Japan	Asia	English	Retrospective cohort	137	CTCAE V4.0	30.66%
24	Dupont R (2019) [92]	France	Europe	English	Retrospective cohort	191	CTCAE V5.0	13.09%
25	Tang SH (2020) [93]	China	Asia	Chinese	Retrospective cohort	110	CTCAE V5.0	50.00%
26	Hosoya K (2020) [94]	Japan	Asia	English	Retrospective cohort and Prospective cohort	224	CTCAE V4.0	18.30%
27	Aso M (2020) [95]	Japan	Asia	English	Retrospective cohort	155	CTCAE V4.0	32.90%
28	Hasan AliO (2020) [96]	Switzerland	Europe	English	Retrospective cohort	40	CTCAE V5.0	40.00%
29	Noguchi S (2020) [97]	Japan	Asia	English	Retrospective cohort	94	CTCAE V4.0	27.66%
30	Amrane K (2020) [98]	French	Europe	English	Retrospective cohort	108	CTCAE V4.0	22.22%
31	Kubo T (2020) [99]	Japan	Asia	English	Retrospective cohort	95	/	26.32%
32	Cortellini A (2020) [100]	Italy, The Netherlands, Switzerland, UK	Europe	English	Retrospective cohort	1010	CTCAE V4.0	9.90%
33	Kichenadasse G (2020) [101]	USA	America	English	Retrospective cohort	1548	CTCAE V4.0	19.77%
34	Shankar B (2020) [102]	NA	NA	English	Retrospective cohort	623	/	7.54%
35	Xie JL (2020) [103]	China	Asia	Chinese	Retrospective cohort	58	CTCAE V4.0	3.45%
36	Ding YL (2020) [104]	China	Asia	Chinese	Retrospective cohort	57	CTCAE V4.0	49.12%
37	Morita R (2020) [105]	Japan	Asia	English	Retrospective cohort	901	CTCAE V4.0	16.76%
38	Gulati N (2020) [106]	USA	America	English	Prospective cohort	184	CTCAE V5.0	28.80%
39	Ye LW (2021) [107]	China	Asia	Chinese	Prospective cohort	88	/	55.68%
40	Sun XD (2021) [108]	China	Asia	Chinese	Retrospective cohort	32	/	6.25%
41	Hu C (2021) [109]	China	Asia	Chinese	Retrospective cohort	36	CTCAE V5.0	11.11%
42	Zhou Y (2021) [110]	China	Asia	Chinese	Retrospective cohort	36	CTCAE	27.78%
43	Zhan LF (2021) [111]	China	Asia	Chinese	Prospective cohort	43	CTCAE V5.0	37.21%
44	Shukla NA (2021) [112]	Indiana	Asia	English	Retrospective cohort	92	/	18.48%
45	Saito Z (2021) [113]	Japan	Asia	English	Retrospective cohort	45	CTCAE V5.0	4.44%
46	Yi WL (2021) [114]	China	Asia	Chinese	Retrospective cohort	109	CTCAE V4.0	33.94%
47	Lv WY (2021) [115]	China	Asia	Chinese	Prospective cohort	42	CTCAE V5.0	16.67%
48	Conde-Estévez D (2021) [116]	Spain	Europe	English	Retrospective cohort	70	CTCAE V5.0	21.43%
49	Huang Y (2021) [117]	Singapore	Asia	English	Retrospective cohort	141	CTCAE V5.0	27.66%
50	Sonehara K (2021) [118]	Japan	Asia	English	Retrospective cohort	80	CTCAE V4.0	6.25%
51	Zhu WJ (2022) [119]	China	Asia	Chinese	Retrospective cohort	174	/	0.57%
52	Zhang XJ (2022) [120]	China	Asia	Chinese	Retrospective cohort	40	CTCAE V5.0	22.50%
53	Shi Y (2022) [121]	China	Asia	English	Retrospective cohort	1905	/	7.61%
54	Yoneda T (2022) [122]	Japan	Asia	English	Retrospective cohort	435	/	10.57%

NA: Not Available; CTCAE V: Common Terminology Criteria for Adverse Events Version; “/”: indicate that specific diagnostic criteria was not adopted in the study.

## Data Availability

All data generated or analyzed during this study are included in the published article and Supplementary Materials.

## Supplementary Materials

The following supporting information can be downloaded at: https://www.mdpi.com/article/10.3390/curroncol32040195/s1, Supplementary File S1: Search strategy of PubMed, Embase, CINAHL, Cochrane, CBM, CNKI, Wanfang; Supplementary File S2: Quality assessment of RCT, nRCT and cohort studies.

## Abbreviations

Immune checkpoint inhibitors	ICIs
Cutaneous immune-related adverse events	cirAEs
Non-small-cell lung cancer	NSCLC
Confidence interval	CI
Randomized controlled trial	RCT
Non-randomized controlled trial	nRCT
CTCAE V	Common Terminology Criteria for Adverse Events Version
