# Supplementary material for: Incidence Rates of Cutaneous Immune-Related Adverse Events in Patients with Lung Cancer: A Systematic Review and Meta-Analysis
Source: Curr Oncol. 2025 Mar 27;32(4):195. doi: 10.3390/curroncol32040195 (PMC12025845; doi:10.3390/curroncol32040195)
Supplement: Supplementary file 1 [file curroncol-32-00195-s001.zip › Supplementary File S1.pdf]

Supplementary File S1-Search strategy

PubMed

#1 Neoplasm[MeSH Terms] Filters: Humans, English

#2 (Immunotherapy[MeSH Terms]) OR (Immune Checkpoint Inhibitors[MeSH Terms]) Filters: Humans, English

#3 skin[MeSH Terms] Filters: Humans, English

#4 ((((neoplas*[Title/Abstract]) OR (tumor[Title/Abstract])) OR(cancer*[Title/Abstract])) OR (malignan*[Title/Abstract])) OR(carcinoma* Title/Abstract]) Filters: Humans, English

#5 (Immunotherapy[Title/Abstract]) OR (Immune Checkpoint*[Title/Abstract]) Filters: Humans, English

#6 (((((skinTitle/Abstract]) OR (immue-related*[Title/Abstract])))OR (irAEs[Title/Abstract])) OR (irCAEs[Title/Abstract])) OR (derma*[Title/Abstract])) OR (cut*[Title/Abstract]) Filters: Humans, English

#7 #1 **OR** #4

#8 #2 **OR** #5

#9 #3 **OR** #6

#10 #7 **AND** # 8**AND** #9

Embase

#1 ‘neoplasm’/exp

#2 "immune checkpoint inhibitor'/exp

#3 "immunotherapy'exp

#4 "skin'/exp

#5 neoplas*ti,ab,kw OR tumor*ti,ab,kw OR cancer*ti,ab,kw OR malignan*ti,ab,kw OR carcinoma*ti,ab,kw

#6 immunotherapy*.ti,ab,kw OR ‘immune checkpoint*’ti.ab,kw

#7 skin:ti,ab,kw OR ‘immue related*’:ti,ab,kw OR iraes:ti,ab,kw OR derma'*’:ti,ab,kw

OR cut*.ti,ab,kw

#8 #1 OR#5

#9 #2 0R#3 OR #6

#10 #4 OR#7

#11 #8 AND #9 AND

#12 #11 AND ‘human’/de

CINAHL

#1 (MM "Neoplasms+")

#2 (MM "Immunotherapy+")

#3 (MM "Immune Checkpoint lnhibitors+")

#4 (MM "skin+")

#5 SU neoplas* OR SU tumor* OR SU cancer OR SU malignan* OR SU carcinoma*

#6 SU Immunotherapy* OR SU lmmune Checkpoint*

#7 SU skin OR SU immue-related* OR SU irAES OR SU irCAES OR SU derma* OR SU cut*

#8 S1 OR S5

#9 S2 OR S3 OR S6

#10 S4 OR S7

#11 S8 AND S9 AND S10

Cochrane

#1 MeSH descriptor: [Neoplasms] explode all trees

#2 MeSH descriptor: [Immunotherapy] explode all trees

#3 MeSH descriptor: [Immune Checkpoint lnhibitors] explode all trees

#4 MeSH descriptor: [Skin] explode all trees

#5 (neoplas*):ti,ab,kw OR (tumor*):ti,ab,kw OR (cancer*):ti,ab,kw(malionan*):ti,ab,kw

#6 (Immunotherapy*):ti,ab,kw OR (Immune Checkpoint)):ti,ab,kw

#7 (skin):ti,ab,kw OR (immue-related*):ti,ab,kw OR (irAEs):ti,ab,kw OR (dema%):ti ab.kw

#8 (cut):ti,ab kw

#9 #1 OR#5

#10 #2 0R#3 OR #6

#11 #4 OR#7 OR#8

#12 #9 AND #10 AND #11

CBM

#1 "癌"[不加权:扩展]

#2 "肿瘤"[不加权:扩展]

#3 "皮肤"[不加权:扩展]

#4 "癌"[常用字段:智能] OR "肿瘤"[常用字段:智能]

#5 "免疫治疗"[常用字段:智能]

#6 "皮肤"[常用字段:智能] OR "不良事件"[常用字段:智能] OR "不良反应"[常用字段智能] OR "毒性"[常用字段:智能] OR "副作用"[常用字段:智能]

#7 (#4) OR (#2) OR (#1)

#8 (#6) OR (#3)

#9 (#8) AND (#7) AND (#5)

CNKI

#1 (篇关摘=癌) OR (篇关摘=肿瘤)

#2 (篇关摘=免疫相关不良事件) OR (篇关摘=免疫相关毒性) OR (篇关摘=免疫相关不良反应) OR (篇关摘=免疫相关副作用) OR (篇关摘=皮肤毒性) OR (篇关摘=皮肤反应)

Wanfang

#1 主题:(("癌") or 主题:("肿瘤")) and 主题:("免疫治疗") and 主题:(("不良事件") or 主题:("毒性") or 主题:("不良反应") or 主题:("副作用") or 主题:("皮肤"))
