# Supplementary material for: Incidence Rates of Cutaneous Immune-Related Adverse Events in Patients with Lung Cancer: A Systematic Review and Meta-Analysis
Source: Curr Oncol. 2025 Mar 27;32(4):195. doi: 10.3390/curroncol32040195 (PMC12025845; doi:10.3390/curroncol32040195)
Supplement: Supplementary file 1 [file curroncol-32-00195-s001.zip › Supplementary File S2.pdf]

Supplementary File S2

Quality assessment of RCT

No	Author (Year)	Title	1	2	3	4	5	6	7	8	9	10	11	12	13
1	Lynch TJ (2012)[24]	Ipilimumab in Combination With Paclitaxel and Carboplatin As First-Line Treatment in Stage IIIB/IV Non-Small-Cell Lung Cancer: Results From a Randomized, Double-Blind, Multicenter Phase II Study.	Y	Y	Y	NA	Y	Y	Y	Y	Y	Y	Y	Y	Y
2	Borghaei H (2015)[25]	Nivolumab versus Docetaxel in Advanced Nonsquamous Non-Small-Cell Lung Cancer.	Y	U	Y	U	U	U	Y	Y	Y	Y	Y	Y	Y
3	Brahmer J (2015)[26]	Nivolumab versus Docetaxel in Advanced Squamous-Cell Non-Small-Cell Lung Cancer.	Y	U	Y	U	N	Y	Y	Y	Y	Y	Y	Y	Y
4	Gettinger SN (2015)[27]	Overall Survival and Long-Term Safety of Nivolumab(Anti-Programmed Death 1 Antibody, BMS-936558,ONO-4538) in Patients With Previously Treated Advanced Non-Small-Cell Lung Cancer.	Y	U	NA	U	U	U	Y	Y	Y	Y	Y	Y	Y
5	Garon EB (2015)[28]	Pembrolizumab for the Treatment of Non-Small-Cell Lung Cancer.	Y	Y	U	U	Y	Y	Y	Y	Y	Y	Y	Y	Y
6	Fehrenbacher L (2016)[29]	Atezolizumab versus docetaxel for patients with previously treated non-small-cell lung cancer (POPLAR): a multicentre, open-label, phase 2 randomised controlled trial.	Y	Y	Y	N	N	N	Y	N	N	Y	Y	Y	Y
7	Langer CJ (2016)[30]	Carboplatin and pemetrexed with or without pembrolizumab for advanced, non-squamous non-small-cell lung cancer: a randomised, phase 2 cohort of the open-label KEYNOTE-021 study.	Y	Y	Y	Y	Y	Y	Y	Y	Y	Y	Y	Y	Y
8	Reck M (2016)[31]	Pembrolizumab versus Chemotherapy for PD-L1 Positive Non-Small-Cell Lung Cancer.	Y	Y	Y	Y	NA	Y	Y	Y	Y	Y	Y	Y	Y
9	Herbst RS (2016)[32]	Pembrolizumab versus docetaxel for previously treated, PD-L1-positive, advanced non-small-cell lung cancer (KEYNOTE-010): a randomised controlled trial.	Y	U	Y	U	U	Y	Y	Y	Y	Y	Y	Y	Y
10	Reck M (2016)[33]	Phase III Randomized Trial of Ipilimumab Plus Etoposide and Platinum Versus Placebo Plus Etoposide and Platinum in Extensive-Stage Small-Cell Lung Cancer.	Y	Y	Y	Y	NA	Y	Y	Y	N	Y	Y	Y	Y
11	Govindan R (2017)[34]	Phase III Trial of Ipilimumab Combined With Paclitaxel and Carboplatin in Advanced Squamous Non-Small-Cell Lung Cancer.	Y	Y	Y	Y	NA	Y	Y	Y	Y	Y	Y	Y	Y
12	Rittmeyer A (2017)[35]	Atezolizumab versus docetaxel in patients with previously treated non-small-cell lung cancer (OAK): a phase 3, open-label, multicentre randomised controlled trial.	Y	NA	Y	NA	NA	NA	Y	Y	Y	Y	Y	Y	Y

**13**	**Antonia SJ (2017)[36]**	**Durvalumab after Chemoradiotherapy in Stage III Non-Small-Cell Lung Cancer.**	**Y**	**Y**	**Y**	**Y**	**U**	**Y**	**Y**	**Y**	**Y**	**Y**	**Y**	**Y**	**Y**
**14**	**Carbone DP (2017)[37]**	**First-Line Nivolumab in Stage IV or Recurrent Non-Small-Cell Lung Cancer.**	**Y**	**Y**	**Y**	**U**	**Y**	**Y**	**Y**	**Y**	**Y**	**Y**	**Y**	**Y**	**Y**
**15**	**Hellmann MD (2017)[38]**	**Nivolumab plus ipilimumab as first-line treatment for advanced non-small-cell lung cancer (CheckMate 012): results of an open-label, phase 1, multicohort study.**	**Y**	**NA**	**Y**	**NA**	**NA**	**NA**	**Y**	**N**	**N**	**Y**	**Y**	**Y**	**Y**
**16**	**Hida T (2018)[39]**	**Atezolizumab in Japanese Patients With Previously Treated Advanced Non Small-Cell Lung Cancer: A Subgroup Analysis of the Phase 3 OAK Study.**	**Y**	**NA**	**Y**	**NA**	**NA**	**NA**	**Y**	**N**	**N**	**Y**	**Y**	**Y**	**Y**
**17**	**Socinski MA (2018)[40]**	**Atezolizumab for First-Line Treatment of Metastatic Nonsquamous NSCLC.**	**Y**	**NA**	**Y**	**NA**	**NA**	**NA**	**Y**	**Y**	**Y**	**Y**	**Y**	**Y**	**Y**
**18**	**Hellmann MD (2018)[41]**	**Nivolumab plus Ipilimumab in Lung Cancer with a High Tumor Mutational Burden.**	**Y**	**Y**	**Y**	**N**	**N**	**Y**	**U**	**Y**	**Y**	**Y**	**Y**	**Y**	**Y**
**19**	**Paz-Ares L (2018)[42]**	**Pembrolizumab plus Chemotherapy for Squamous Non-Small-Cell Lung Cancer.**	**Y**	**Y**	**Y**	**Y**	**N**	**Y**	**Y**	**Y**	**Y**	**Y**	**Y**	**Y**	**Y**
**20**	**Gandhi L (2018)[43]**	**Pembrolizumab plus Chemotherapy in Metastatic Non-Small-Cell Lung Cancer.**	**Y**	**Y**	**Y**	**Y**	**Y**	**Y**	**Y**	**Y**	**Y**	**Y**	**Y**	**Y**	
**21**	**Hellmann MD (2019)[44]**	**Nivolumab plus Ipilimumab in Advanced Non-Small Cell Lung Cancer.**	**Y**	**Y**	**Y**	**Y**	**U**	**Y**	**Y**	**N**	**N**	**Y**	**Y**	**Y**	**Y**
**22**	**Wu YL(2019)[45]**	**Nivolumab Versus Docetaxel in a Predominantly Chinese Patient Population With Previously Treated Advanced NSCLC: CheckMate 078 Randomized Phase III Clinical Trial.**	**Y**	**NA**	**Y**	**N**	**N**	**Y**	**Y**	**Y**	**Y**	**Y**	**Y**	**Y**	**Y**
**23**	**Gubens MA (2019)[46]**	**Pembrolizumab in combination with ipilimumab as second-line or later therapy for advanced non-small-cell lung cancer: KEYNOTE-021 cohorts D and H.**	**Y**	**NA**	**Y**	**NA**	**NA**	**Y**	**Y**	**Y**	**Y**	**Y**	**Y**	**Y**	**Y**
**24**	**Mok TSK (2019)[47]**	**Pembrolizumab versus chemotherapy for previously untreated, PD-L1-expressing, locally advanced or metastatic non-small-cell lung cancer (KEYNOTE-042): a randomised, open-label, controlled, phase 3 trial.**	**Y**	**N**	**Y**	**N**	**N**	**Y**	**Y**	**Y**	**Y**	**Y**	**Y**	**Y**	**Y**
**25**	**Reck M (2019)[48]**	**Updated Analysis of KEYNOTE-024:Pembrolizumab Versus Platinum-Based Chemotherapy for Advanced Non-Small-Cell Lung Cancer With PD-L1 Tumor Proportion Score of 50% or Greater.**	**Y**	**N**	**Y**	**N**	**N**	**Y**	**Y**	**Y**	**Y**	**Y**	**Y**	**Y**	**Y**
**26**	**Li K (2021)[49]**	**Explore the clinical effects of PD-1/PD-L1 inhibitors in the treatment of non-small cell lung cancer.**	**Y**	**U**	**Y**	**U**	**U**	**Y**	**U**	**Y**	**Y**	**Y**	**Y**	**Y**	**Y**
**27**	**Bai Y (2021)[50]**	**PD-1 inhibitor combined with chemotherapy in preoperative neoadjuvant treatment of stage Ⅲ non-small cell lung cancer: A randomized controlled trial.**	**Y**	**U**	**Y**	**U**	**U**	**Y**	**U**	**Y**	**Y**	**Y**	**Y**	**Y**	**Y**

28	Lv Y (2021)[51]	The short-term efficacy and safety of camrelizumab combined with concurrent radiochemotherapy in non-squamous NSCLC patients with driver gene-negative and brain metastases.	Y	U	Y	U	U	Y	U	Y	Y	Y	Y	Y	Y
29	He M (2021)[52]	Application Effect of Predictive Nursing in Immunotherapy of Lung Cancer Patients.	Y	U	Y	NA	NA	Y	U	Y	Y	Y	Y	Y	Y
30	Li LR(2022)[53]	The application effect of self-care in lung cancer immunotherapy.	Y	U	Y	NA	NA	Y	U	Y	Y	Y	Y	Y	Y

Quality assessment of nRCT

No	Author (Year)	Title	1	2	3	4	5	6	7	8	9
1	Rizvi NA (2015)[54]	Activity and safety of nivolumab, an anti-PD-1 immune checkpoint inhibitor, for patients with advanced, refractory squamous non-small-cell lung cancer (CheckMate 063): a phase 2, single-arm trial.	Y	NA	NA	N	Y	Y	Y	Y	Y
2	Nishio M (2016)[55]	Multicentre phase II study of nivolumab in Japanese patients with advanced or recurrent non-squamous non-small cell lung cancer.	Y	NA	NA	N	Y	Y	Y	Y	Y
3	Antonia SJ (2016)[56]	Nivolumab alone and nivolumab plus ipilimumab in recurrent small-cell lung cancer (CheckMate 032): a multicentre, open-label, phase 1/2 trial.	Y	Y	Y	Y	Y	N	Y	Y	Y
4	Gettinger S (2016)[57]	Nivolumab Monotherapy for First-Line Treatment of Advanced Non-Small-Cell Lung Cancer.	Y	NA	NA	N	Y	Y	Y	Y	Y
5	Goldberg SB (2016)[58]	Pembrolizumab for patients with melanoma or non-small-cell lung cancer and untreated brain metastases: Early analysis of a non-randomised, open-label, phase 2 trial.	Y	Y	Y	Y	Y	Y	Y	Y	Y
6	Antonia S (2016)[59]	Safety and antitumour activity of durvalumab plus tremelimumab in non-small-cell lung cancer: a multicentre, phase 1b study.	Y	Y	Y	Y	Y	Y	Y	Y	Y
7	Hida T (2017)[60]	Efficacy and safety of nivolumab in Japanese patients with advanced or recurrent squamous non-small cell lung cancer.	Y	NA	Y	N	Y	Y	Y	Y	Y
8	Peters S (2017)[61]	Phase II Trial of Atezolizumab As First-Line or Subsequent Therapy for Patients With Programmed Death-Ligand 1-Selected Advanced Non-Small-Cell Lung Cancer (BIRCH).	Y	Y	Y	Y	Y	Y	Y	Y	Y
9	Lee J S (2018)[62]	Nivolumab in advanced non-small-cell lung cancer patients who failed prior platinum-based chemotherapy.	Y	Y	Y	Y	Y	Y	Y	Y	Y
10	Nishio M (2019)[63]	KEYNOTE-025: Phase 1b study of pembrolizumab in Japanese patients with previously treated programmed	Y	NA	NA	N	Y	Y	Y	Y	Y

		death ligand 1-positive advanced non-small-cell lung cancer.									
11	Li JW (2019)[64]	The single-center analysis of the efficacy and safety of nivolumab monoclonal antibody in advanced non-small cell lung cancer.	Y	NA	NA	N	Y	Y	Y	Y	Y
12	Wang R (2020)[65]	Observation and nursing of adverse reactions in the treatment of advanced lung cancer with anti-PD-1 monoclonal antibody.	Y	NA	NA	N	Y	Y	Y	Y	Y
13	Deng WJ (2020)[66]	Efficacy Observation on effectiveness and safety of PD-1 monoclonal antibody Pembrolizumab in 28 patients with advanced lung cancer.	Y	NA	NA	N	Y	Y	Y	Y	Y
14	Gan WL (2021)[67]	A research on symptoms of skin toxicity induced by immunotherapy in patients with lung cancer.	Y	Y	Y	Y	Y	Y	Y	Y	Y
15	Bai Y (2022)[68]	Clinical study of PD-1 monoclonal antibody combined with chemotherapy in the preoperative neoadjuvant treatment of stage ⅢA non-small cell lung cancer.	Y	Y	Y	Y	Y	Y	Y	Y	Y

Quality assessment of cohort studies

No	Author (Year)	Title	1	2	3	4	5	6	7	8	9	10	11
1	Hasan AliO (2016)[69]	Characterization of nivolumab-associated skin reactions in patients with metastatic non-small cell lung cancer.	Y	NA	Y	Y	Y	Y	Y	Y	Y	NA	Y
2	Teraoka S (2017)[70]	Early Immune-Related Adverse Events and Association with Outcome in Advanced Non-Small Cell Lung Cancer Patients Treated with Nivolumab: A Prospective Cohort Study.	Y	NA	Y	Y	Y	Y	Y	Y	Y	NA	Y
3	Hui R (2017)[71]	Pembrolizumab as first-line therapy for patients with PD-L1-positive advanced non-small cell lung cancer: a phase 1 trial.	Y	Y	Y	Y	Y	Y	Y	Y	Y	U	Y
4	Haratani K (2018)[72]	Association of Immune-Related Adverse Events With Nivolumab Efficacy in Non-Small-Cell Lung Cancer.	Y	NA	Y	Y	Y	Y	Y	Y	Y	NA	Y
5	Min Lee CK (2018)[73]	Characterization of dermatitis after PD-1 PD-L1 inhibitor therapy and association with multiple oncologic outcomes A retrospective case-control study.	Y	Y	Y	Y	Y	Y	Y	Y	Y	U	Y
6	Sato K (2018)[74]	Correlation between immune-related adverse events and efficacy in non small cell lung cancer treated with nivolumab.	NA	NA	Y	Y	Y	Y	Y	Y	Y	U	Y

7	Fiorica F (2018)[75]	Immune Checkpoint Inhibitor Nivolumab and Radiotherapy in Pretreated Lung Cancer Patients Efficacy and Safety of Combination.	Y	Y	Y	Y	Y	Y	Y	Y	Y	NA	Y
8	Owen DH (2018)[76]	Incidence, Risk Factors, and Effect on Survival of Immune-related Adverse Events in Patients With Non-Small-cell Lung Cancer.	Y	NA	Y	Y	Y	Y	Y	Y	Y	NA	Y
9	Sabatier R (2018)[77]	Nivolumab in routine practice for older patients with advanced or metastatic non-small cell lung cancer.	Y	NA	Y	U	U	Y	Y	Y	Y	NA	Y
10	Schouten RD (2018)[78]	Real life experience with nivolumab for the treatment of non-small cell lung carcinoma Data from the expanded access program and routine clinical care in a tertiary cancer centre-The Netherlands Cancer Institute.	Y	NA	Y	Y	Y	Y	Y	Y	Y	NA	Y
11	Areses Manrique MC (2018)[79]	Real world data of nivolumab for previously treated non-small cell lung cancer patients: a Galician lung cancer group clinical experience.	Y	NA	Y	Y	Y	Y	Y	Y	Y	NA	Y
12	Grossi F (2018)[80]	Use of nivolumab in elderly patients with advanced squamous non-small-cell lung cancer: results from the Italian cohort of an expanded access programme.	Y	NA	Y	Y	Y	Y	Y	Y	Y	NA	Y
13	Akano Y (2019)[81]	Analysis of Pleiotropic Effects of Nivolumab in Pretreated Advanced or Recurrent Non-small Cell Lung Cancer Cases.	Y	NA	Y	Y	Y	Y	Y	Y	Y	NA	Y
14	Lee YJ (2019)[82]	Characterization and Prognostic Significance of Cutaneous Adverse Events to Anti-Programmed Cell Death-1 Therapy.	Y	Y	Y	Y	Y	Y	Y	Y	Y	U	Y
15	Krefting F (2019)[83]	Clinical Experience of Immunotherapy Treatment: Efficacy and Toxicity Analysis of the Compassionate Use Program of Nivolumab in Patients with Advanced Squamous Cell Non-Small Cell Lung Cancer.	Y	NA	Y	Y	Y	Y	Y	Y	Y	NA	Y
16	Ahn BC (2019)[84]	Comprehensive analysis of the characteristics and treatment outcomes of patients with non-small cell lung cancer treated with anti-PD-1 therapy in real-world practice.	Y	NA	Y	Y	Y	Y	Y	Y	Y	NA	Y
17	Cortellini A (2019)[85]	Correlations Between the Immune-related Adverse Events Spectrum and Efficacy of Anti-PD1 Immunotherapy in NSCLC Patients.	Y	NA	Y	Y	Y	Y	Y	Y	Y	NA	Y
18	Ksienski D (2019)[86]	Efficacy of Nivolumab and Pembrolizumab in Patients With Advanced Non-Small-Cell Lung Cancer Needing Treatment Interruption Because of Adverse Events: A Retrospective Multicenter Analysis.	Y	Y	Y	Y	Y	Y	Y	Y	Y	NA	Y
19	Muchnik E (2019)[87]	Immune Checkpoint Inhibitors in Real-World Treatment of Older Adults with Non-Small Cell Lung Cancer.	Y	NA	Y	Y	Y	Y	Y	Y	Y	NA	Y
20	Ricciuti B (2019)[88]	Impact of immune-related adverse events on survival in patients with advanced non-small cell lung cancer	Y	NA	Y	Y	Y	Y	Y	Y	Y	NA	Y

		treated with nivolumab: long-term outcomes from a multi-institutional analysis.											
21	Ksienski D (2019)[89]	Pembrolizumab for advanced non-small cell lung cancer: Efficacy and safety in everyday clinical practice.	Y	NA	Y	Y	Y	Y	Y	Y	Y	NA	Y
22	Pavan A (2019)[90]	Peripheral Blood Markers Identify Risk of Immune‐Related Toxicity in Advanced Non‐Small Cell Lung Cancer Treated with Immune-Checkpoint Inhibitors.	Y	NA	Y	Y	Y	Y	Y	Y	Y	NA	Y
23	Toi Y (2019)[91]	Profiling Preexisting Antibodies in Patients Treated With Anti-PD-1 Therapy for Advanced Non-Small Cell Lung Cancer.	Y	Y	Y	Y	Y	Y	Y	Y	Y	U	Y
24	Dupont R (2019)[92]	The prognostic impact of immune-related adverse events during anti-PD1 treatment in melanoma and non-small-cell lung cancer: a real-life retrospective study.	Y	Y	Y	Y	Y	Y	Y	Y	Y	NA	Y
25	Tang SH (2020)[93]	Longitudinal study of skin toxicity caused by immunotherapy in patients with non-small cell lung cancer.	NA	NA	Y	U	U	Y	Y	Y	Y	U	Y
26	Hosoya K (2020)[94]	Association Between Early Immune-related Adverse Events and Clinical Outcomes in Patients With Non-Small Cell Lung Cancer Treated With Immune Checkpoint Inhibitors.	N	NA	Y	Y	Y	Y	Y	Y	Y	U	Y
27	Aso M (2020)[95]	Association Between Skin Reaction and Clinical Benefit in Patients Treated with Anti-Programmed Cell Death 1 Monotherapy for Advanced Non-Small Cell Lung Cancer.	Y	Y	Y	Y	Y	Y	Y	Y	Y	U	Y
28	Hasan AliO (2020)[96]	BP180-specific IgG is associated with skin adverse events, therapy response, and overall survival in non-small cell lung cancer patients treated with checkpoint inhibitors.	Y	Y	Y	Y	Y	Y	Y	Y	Y	NA	Y
29	Noguchi S (2020)[97]	Correlation of Immune-Related Adverse Events and Effects of Pembrolizumab Monotherapy in Patients with Non-Small Cell Lung Cancer.	Y	NA	Y	Y	Y	Y	Y	Y	Y	NA	Y
30	Amrane K (2020)[98]	First-line pembrolizumab for non-small cell lung cancer patients with PD-L1 ≥50% in a multicenter real-life cohort: The PEMBREIZH study.	Y	NA	Y	Y	Y	Y	Y	Y	Y	NA	Y
31	Kubo T (2020)[99]	Immune checkpoint inhibitor efficacy and safety in older non-small cell lung cancer patients.	Y	NA	Y	Y	Y	Y	Y	Y	Y	NA	Y
32	Cortellini A (2020)[100]	Immune-related Adverse Events of Pembrolizumab in a Large Real-world Cohort of Patients With NSCLC With a PD-L1 Expression ≥ 50% and Their Relationship With Clinical Outcomes.	Y	NA	Y	Y	Y	Y	Y	Y	Y	NA	Y
33	Kichenadasse G (2020)[101]	Multiorgan Immune-Related Adverse Events During Treatment With Atezolizumab.	NA	NA	Y	Y	Y	Y	Y	Y	Y	U	Y
34	Shankar B (2020)[102]	Multisystem Immune-Related Adverse Events associatted With Immune Checkpoint Inhibitors for Treatment	Y	Y	Y	Y	Y	Y	Y	Y	Y	NA	Y

		of Non-Small Cell Lung Cancer.											
35	Xie JL (2020)[103]	Efficacy and safety of PD-1/PD-L1 immune checkpoint inhibitors in KRAS-mutant advanced non-small cell lung cancer.	Y	N	Y	Y	Y	Y	Y	Y	NA	NA	Y
36	Ding YL (2020)[104]	Investigation on the clinical application and safety of Anti-PD-1 in the treatment of NSCLC based on real world.	Y	Y	Y	Y	Y	Y	Y	Y	Y	NA	Y
37	Morita R (2020)[105]	Real-world effectiveness and safety of nivolumab in patients with non-small cell lung cancer: A multicenter retrospective observational study in Japan.	Y	Y	Y	Y	Y	Y	Y	Y	Y	NA	Y
38	Gulati N (2020)[106]	Revisiting the association between skin toxicity and better response in advanced cancer patients treated with immune checkpoint inhibitors.	Y	N	Y	Y	Y	Y	Y	Y	Y	NA	Y
39	Ye LW (2021)[107]	Investigation report on skin toxicity caused by Immunotherapy in non small cell lung cancer patients.	NA	NA	Y	Y	Y	Y	Y	Y	Y	NA	Y
40	Sun XD (2021)[108]	The efficacy and safety of domestic immune checkpoint inhibitors combined with chemotherapy in the treatment of advanced non-small cell lung cancer.	Y	Y	Y	U	U	Y	Y	Y	Y	U	N
41	Hu C (2021)[109]	Clinical analysis of immune checkpoint inhibitors in the treatment of advanced lung cancer with immune related toxicity.	NA	NA	Y	Y	Y	Y	Y	Y	Y	U	Y
42	Zhou Y (2021)[110]	Observation of the therapeutic effect of immune checkpoint inhibitors on advanced non-small cell lung cancer.	Y	Y	Y	Y	Y	Y	Y	Y	Y	U	Y
43	Zhan LF (2021)[111]	Clinical observation of Sintilimab plus platinum based chemotherapy as first-line treatment for metastatic non-small cell lung cancer (NSCLC).	NA	NA	Y	U	U	Y	Y	Y	Y	U	Y
44	Shukla NA (2021)[112]	Association of Immune-Related Adverse Events and Efficacy Outcomes With Consolidation Pembrolizumab After Chemoradiation in Patients With Inoperable Stage III Non-Small-Cell Lung Cancer.	Y	N	Y	Y	Y	Y	Y	Y	Y	NA	Y
45	Saito Z (2021)[113]	Efficacy and safety of immune checkpoint inhibitors in patients with non-small cell lung cancer aged 80 years or older.	Y	Y	Y	Y	Y	Y	Y	Y	Y	NA	Y
46	Yi WL (2021)[114]	Analysis of immune-related adverse events and its correlation with efficacy of anti-PD-1 monotherapy in advanced non-small cell lung cancer.	Y	Y	Y	Y	Y	Y	Y	Y	Y	NA	Y
47	Lv WY (2021)[115]	Analysis of the influencing factors of adverse drug reactions in PD-1 immunotherapy for non-small cell lung cancer.	Y	Y	Y	Y	Y	Y	Y	Y	Y	U	Y

48	Conde-Estévez D (2021)[116]	Prognostic factors and effect on survival of immune-related adverse events in patients with non-small-cell lung cancer treated with immune checkpoint blockage.	Y	NA	Y	Y	Y	Y	Y	Y	Y	NA	Y
49	Huang Y (2021)[117]	Risk factors for immune-related adverse events from anti-PD-1 or anti-PD-L1 treatment in an Asian cohort of non small cell lung cancer patients.	NA	NA	Y	Y	Y	Y	Y	Y	Y	U	Y
50	Sonehara K (2021)[118]	The Role of Immune-Related Adverse Events in Prognosis and Efficacy Prediction for Patients with Non-Small Cell Lung Cancer Treated with Immunotherapy: A Retrospective Clinical Analysis.	Y	NA	Y	Y	Y	Y	Y	Y	Y	NA	Y
51	Zhu WJ (2022)[119]	Real-world study on the efficacy and prognostic predictive biomarker of patients with metastatic non-small cell lung cancer treated with programmed death-1/programmed death ligand 1 inhibitors.	Y	Y	Y	Y	Y	Y	Y	Y	Y	U	Y
52	Zhang XJ(2022)[120]	Effect of Camrelizumab combined with chemotherapy in the first-line treatment of advanced lung adenocarcinoma without sensitive gene mutation.	Y	Y	Y	NA	NA	Y	Y	Y	Y	U	Y
53	Shi Y(2022)[121]	Immune checkpoint inhibitor-related adverse events in lung cancer: Real-world incidence and management practices of 1905 patients in China.	NA	NA	Y	Y	Y	Y	Y	Y	Y	U	Y
54	Yoneda T (2022)[122]	Long-Term Survival of Patients With Non-Small Cell Lung Cancer Treated With Immune Checkpoint Inhibitor Monotherapy in Real-World Settings.	NA	NA	Y	Y	Y	Y	Y	Y	Y	U	Y
